# DEVELOPING A SOCIOLOGY OF COVID-19: CHALLENGES AND PRIORITIES FOR SOCIAL GERONTOLOGY

**DOI:** 10.1093/geroni/igae098.1972

**Published:** 2024-12-31

**Authors:** Chris Phillipson

**Affiliations:** University of Manchester, Manchester, England, United Kingdom

## Abstract

Much of the discussion about the impact of COVID-19 has been around its biomedical and epidemiological dimensions, with analysis focusing on the origins of the disease, the development of vaccines, and lessons to be learnt to protect against future pandemics. But the sociological issues arising from COVID-19 are important as well. Pandemics are viewed through particular social values and cultural perspectives, reflect economic and social inequalities in their development and impact, and are managed through socially organized forms of care and support. People will almost certainly view themselves and their society in a different way during and following a pandemic: diminished through the loss of partners and friends; strengthened through coming together at a time of crisis; or, conversely, weakened through a sense that some have suffered more than others. COVID-19 affected groups in different ways: by age, location, occupation, quality of housing, and ethnic group. But the highly unequal effects of the pandemic were often overlooked, or just assumed to be an inevitable part of ‘living with the virus’. The paper argues that research in social gerontology over-emphasized the impact of ageism on the experience of COVID-19, neglecting the wider social fissures and economic and racial injustices that unleashed the pandemic. The paper will review priorities for social gerontology in developing work on COVID-19, including understanding new inequalities which have emerged coming out of the pandemic, and developing a sociologically-informed public health working directly with groups in low-income communities to develop partnerships to tackle ongoing and future pandemics.

